# Understanding the Use of Electronic Means to Seek Personal Health Information Among Adults in the United States

**DOI:** 10.7759/cureus.11190

**Published:** 2020-10-27

**Authors:** Noara Alhusseini, Jim E Banta, Jisoo Oh, Susanne Montgomery

**Affiliations:** 1 Public Health, Alfaisal University, Riyadh, SAU; 2 Public Health, Loma Linda University, Loma Linda, USA

**Keywords:** health information, internet, electronics, health belief model, depression, hints

## Abstract

In this study, we explored who is most likely to use electronic means to seek health information and why; our research was guided by the Health Belief Model (HBM). We used the National Cancer Institute’s Health Information Trends Survey (HINTS) dataset for 2017 and 2018 (n=6,697). We found that 67.5% of US adults used electronic means to seek health information and that females (52.4%), non-Hispanic whites (63.8%), those with at least some college education (76.5%), and those with a household income of at least $50,000 per year (58.3%) were most likely to do so. Respondents reporting depression were 42% more likely to use electronic means to seek health information, suggesting that stigma about mental health may direct people with depression to seek online information to avoid face-to-face communication. Using a tablet to track progress on a health-related goal [odds ratio (OR)=2.38, p<0.0001], and using a tablet to make a decision about treating an illness (OR=6.00, p<0.0001) were highly associated with seeking electronic health information. As the internet remains largely unregulated, this suggests that health systems link their patients to trustworthy resources for preventive and treatment-related information, since many already engage in internet-guided health information-seeking.

## Introduction

The internet is comprised of interconnected, global networks that facilitate communication and access to a variety of information [1]. This is achieved with a broad range of channels including websites, blogs, and social media networks that provide user-generated content [2]. Over three billion adults use the internet worldwide for communication and 90% of them use it to access information [3,4]. Information-seeking refers to the purposive search for information to satisfy a goal [5]. Seeking online health information is the third most common online activity among the US population [4] with 74% of adults using the web and 80% of them searching for health-related information [6], mostly about a specific disease (66%) or treatment plan (56%) [4].

The internet is considered a rich source of information and has a large online medical resource, containing over 100,000 health-related websites. It contains formal and informal sources allowing for health information dissemination in a fast, easy, and accessible way [5]. With the development of electronic health platforms, a plethora of new opportunities for individuals to communicate, access, and exchange health-related information have emerged. Many electronic sources such as mobile devices, computers, tablets, smartphones, and electronic book devices enable users to connect to the internet to seek health information [7].

More recently, the internet has become a vital source for individuals who seek health-related information [8]. There are many factors that encourage individuals to seek health-related information. The increased availability of online medical information, its cost-effectiveness, and the ability to tailor the information based on users’ needs highly motivate internet users to use the web to seek health-related information. In addition, individuals with an increased desire to take more responsibility for their health are attracted to the internet because it allows finding instant information that may guide their behaviors and lifestyles. People who use the internet to look up information are called internet surfers. They consider the internet a useful instrument to increase health awareness by finding support from healthcare providers and other users who share medical experiences.

Seeking health information 

To better understand individuals’ behaviors when seeking online health information, researchers have examined patients’ behaviors. For example, Caiata-Zufferey et al. (2010) confirmed that patients often use the internet before and after a medical encounter. Before a consultation, patients use online sources to assess their needs, choose the right healthcare provider, and prepare for the consultation. After a consultation, they go online to understand their diagnosis, treatment plans, and look for alternative medical options. Seeking online health information can also be driven by patients’ dissatisfaction with the consultation or a desire to avoid bothering healthcare providers [9].

For many, the internet is considered a useful tool to research information because it has many resources that match the needs of diverse consumers. Internet users seek online support for medical-related information. People use the web to search for certain medical conditions to increase knowledge, find emotional support, and exchange experiences. Users are highly motivated because they can limit their search and find answers rapidly to health-related queries and concerns. It also allows access and contributions from all users equally, regardless of background, race, or age. Every user gets a chance to search freely and express his/her opinion without discrimination [2]. Therefore, online consumers consider the internet a popular source of health information. The vastness of health information on the internet attracts consumers to use it, especially in the setting of a global push for consumer-focused healthcare [10].

There are also a number of pitfalls to seeking health information on the internet. With few checks and balances and a general lack of universal quality requirements for publishing health-related content, the internet can provide unreliable, inconsistent, and at times misleading medical information [2,10,11]. The volume of available health information on the internet can result in information overload. In addition, the excessive volume of irrelevant information obtained from complex search engines and the technical language involved often cause confusion in lay consumers [10].

Utilization of theory in the context of health information-seeking on the internet 

It is well established that interventions guided by theories are more effective in promoting change. Since many consumers already use the internet as part of personally guided efforts to consider behavior change-related decision-making, it would suggest that taking a theoretical lens to the issue may be of benefit. The Health Belief Model (HBM) has been noted to be useful in advancing the efficacy of intervention both online and offline [12]. Using a theoretical lens like that of the HBM may assist in this regard as the HBM could aid in advancing the efficacy of online and offline health promotion intervention. According to Glanz et al. (2015), individuals are likely to engage in a health-related behavior if they think that they are susceptible to a condition, see the condition as serious, believe that there is benefit in that behavior (such as information-seeking via the internet), that the perceived costs or barriers are outweighed by the benefits, and believe that they can do this efficaciously [13].

Purpose of the study

Seeking health information on the internet and social media has become common among adults in the United States. Researchers are still uncertain about using online health interventions and changes in health behaviors. In addition, there is limited research exploring the use of electronic methods to seek health information from a theoretical framework perspective to understand healthy behaviors to manage chronic diseases. There is a clear gap in the literature on a national level to understand and explore the phenomenon of using the internet and social media to seek health information. 

Since there is a lack of research data about using electronic means to look for health information, we used variables aligned with conceptual components of the HBM to explore using electronic means to seek health information including perceived susceptibility, perceived severity, perceived benefits, and perceived self-efficacy.

Research question

Our study focused on addressing the following question: among adults in the United States, do the components of the HBM as measured by perceived susceptibility, severity, benefits, and self-efficacy predict using electronic devices to seek health information within the past 12 months?

Sub-Questions

This will be accomplished by answering the following sub-questions: does perceived susceptibility predict using electronic devices to seek health information within the past 12 months among adults in the United States? Does perceived severity predict using electronic devices to seek health information within the past 12 months among adults in the United States? Do perceived benefits predict using electronic devices to seek health information within the past 12 months among adults in the United States? Does perceived self-efficacy to take care of one’s health predict using electronic devices to seek health information within the past 12 months among adults in the United States? What is the relative contribution of each conceptual element of the HBM in explaining using electronic means to look for health information?

## Materials and methods

The Health Information Trends Survey (HINTS) dataset was used in this study. A secondary analysis was conducted using HINTS 5, cycle 1 of 2017, and HINTS 5, cycle 2 of 2018. The HINTS is a nationally representative survey that has been administered by the National Cancer Institute (NCI) since 2003. It is freely available for public use. The Institutional Review Board at Loma Linda University approved this study. The target population consisted of civilian non-institutionalized adults aged 18 or older in the United States. The HINTS program collects data on the needs for, access to, and use of health-related information and health-related behaviors and knowledge among Americans. The data were collected using a self-administered mail questionnaire. The dataset 5 cycle 1 of 2017 consisted of 3,285 respondents and the dataset of 2018 consisted of 3,504 respondents. The two datasets were merged to obtain a total of 6,697 respondents. Ninety-two surveys were dropped due to missing data for the outcome measure.

HINTS exclusion criteria included adolescents, children, homeless people, and institutionalized populations. Every sampled participant who completed the survey received a full sample weight, which was used to calculate population and subpopulation estimates. Computation of the full-sample weights comprised the following phases: calculating household level base weights, adjusting for household nonresponse, calculating person-level initial weights, and calibrating the person-level weights to population counts. The replicate weights were calculated using the jackknife replication method [14].

Our outcome measure was “using electronic means to seek health information” measured by addressing the following question: “In the past 12 months, have you used a computer, smartphone, or other electronic means to do the following: look for health or medical information for yourself? (Yes/no).” We then reviewed the existing standardized HINTS survey to identify variables we could align with components of the HBM. For perceived susceptibility, we used the following question: “In general, would you say your health is?” The answer choices included excellent, very good, good, fair, and poor. Six variables were assigned to perceived severity. The survey questions were: “Has your doctor or other health professional ever told you that you had any of the following medical conditions: diabetes or high blood sugar; high blood pressure or hypertension; a heart condition such as heart attack, angina, or congestive heart failure; chronic lung disease, asthma, emphysema, or chronic bronchitis; arthritis or rheumatism; and depression or anxiety disorder.” The answer choices for the questions were (yes/no). For perceived benefits, two variables were assigned. The first question was as follows: “Has your tablet or smartphone helped you track progress on a health-related goal such as quitting smoking, losing weight, or increasing physical activity?” and the second question was, “Has your tablet or smartphone helped you make a decision about how to treat an illness or condition?” The answer choices were (yes/no).

For self-efficacy, one variable was identified: “Overall, how confident are you about your ability to take good care of your health?” The answer choices included: completely confident, very confident, somewhat confident, a little confident, and not confident at all. Demographic variables included age with five categories: gender (male, female), race/ethnicity (non-Hispanic white, non-Hispanic black, Hispanic, non-Hispanic other); education with five categories: less than high school, high school graduate, some college, bachelor’s degree, and post-baccalaureate degree; household income with four categories: $0-19,999, $20,000-49,999, $50,000-99,999, and $100,000 or more; and employment with three categories: employed, retired, and other.

We conducted a series of chi-squared tests to study the relationship between electronic means to seek health information and demographics (Table 1). We then created five models for logistic regression analysis to determine the model fit. Model 1 explored the effect of demographics on the outcome variable, using electronic means to seek health information. Model 2 included the outcome and the first conceptual component of the HBM, perceived susceptibility. Model 3 included the outcome and the second conceptual component, perceived severity. Model 4 included the outcome and the third conceptual component, perceived benefits. Model 5 included the outcome and the fourth conceptual component, perceived self-efficacy. Then, a final model was created with only those variables that were found statistically significant in the previous five models.

## Results

As shown in Table 1, 6,697 respondents completed the survey, representing an estimated annual 245.8 million adults in the United States. More than two-thirds of the respondents (67.5%) had used electronic means to seek health information for themselves. Among those who did, the majority (58.8%) were between 35 and 64 years of age; 52.4% were female, most were non-Hispanic whites (63.8%), and most had at least some college education (76.5%). Household income for the majority (58.3%) of personal health information seekers was at least $50,000 per year and the majority (63%) of them were employed. Those who did not use electronic means to seek personal health information were older individuals: 64.2% were 50 years of age or older; 51% were high school graduates or less, and 52.6% had a household income of less than $50,000 per year. All comparisons were statistically significant (p<0.0001).

**Table 1:** Survey-weighted sociodemographic characteristics of HINTS respondents, 2017-2018 (n=6,697) HINTS: Health Information Trends Survey

Using electronic means to seek health information	Yes, n (%)	No, n (%)	P-value
Total	4,523	2,174	
Age (years)			<0.0001
18-34	669 (26.3)	97 (11.9)	
35-49	1,082 (30.5)	222 (18.7)	
50-64	1,522 (28.3)	635 (32.5)	
65-74	836 (9.3)	553 (14.5)	
75+	320 (3.9)	545 (17.2)	
Missing	93 (1.7)	122 (5.2)	
Gender			<0.0001
Male	1,741 (46.7)	923 (51.8)	
Female	2,727 (52.4)	1,189 (45.5)	
Missing	55 (0.9)	62 (2.7)	
Race/ethnicity			<0.0001
Non-Hispanic white	2,788 (63.8)	1,035 (52.0)	
Non-Hispanic black	513 (9.1)	325 (11.1)	
Hispanic	551 (13.8)	323 (16.5)	
Non-Hispanic other	385 (8.4)	121 (5.9)	
Missing	286 (4.8)	370 (14.5)	
Education			<0.0001
Less than high school	161 (5.3)	315 (16.7)	
High school graduate	583 (17.0)	644 (34.3)	
Some college	1,318 (37.5)	639 (31.9)	
Bachelor’s degree	1,407 (24.0)	318 (8.8)	
Post-baccalaureate	988 (15.0)	178 (5.6)	
Missing	66 (1.2)	80 (2.7)	
Household income			<0.0001
$0-19,999	517 (11.7)	599 (25.7)	
$20,000-49,999	1,018 (22.4)	604 (26.9)	
$50,000-99,999	1,379 (30.5)	431 (21.6)	
$100,000 or more	1,240 (27.8)	194 (11.9)	
Missing	369 (7.5)	346 (14.0)	
Employment			<0.0001
Employed	2,597 (63.0)	658 (39.1)	
Retired	1,109 (13.5)	937 (28.5)	
Other	696 (21.1)	436 (25.5)	
Missing	121 (2.4)	143 (6.9)	

Table 2 presents the bi-variable chi-squared test of using electronic means to seek health information and the conceptually aligned HBM variables. Most respondents reported at least very good health (51.1%) with no chronic conditions. Using electronic means to make a decision about treating an illness (42.1%) or using electronic means to track progress on a health-related goal (46.8%) was significantly related to seeking electronic health information (p<0.0001). While most reported being at least somewhat confident in taking care of themselves (94.9%), this was not significantly related to seeking electronic health information. For perceived severity, i.e. having a serious health diagnosis and its relationship to seeking health information, all chronic diseases except lung disease were significantly related to seeking electronic health information. However, those with hypertension or a mental health diagnosis were most likely to use the internet to seek health information: 32.5% and 24.7% respectively.

**Table 2:** Survey-weighed descriptive measures of the Health Belief Model conceptual framework, stratified by using electronic means to seek health information

Using electronic means to seek health information	Yes, n (%)	No, n (%)	P-value
Total	4,523	2,174	
Perceived susceptibility			0.0003
General health			
Excellent	562 (13.1)	208 (13.1)	
Very good	1,734 (38.0)	693 (31.6)	
Good	1,539 (34.7)	774 (33.3)	
Fair	545 (11.6)	392 (17.0)	
Poor	98 (2.0)	82 (4.1)	
Missing	37 (0.6)	24 (0.8)	
Perceived severity			<0.0001
Diabetes/high blood sugar			
Yes	839 (14.5)	530 (21.8)	
No	3,610 (84.1)	1,571 (75.0)	
Missing	74 (1.4)	73 (3.2)	
Hypertension/high blood pressure			<0.0001
Yes	1,840 (32.5)	1,158 (44.7)	
No	2,614 (66.2)	945 (52.4)	
Missing	69 (1.3)	71 (2.9)	
Heart condition			0.0003
Yes	364 (6.1)	295 (10.2)	
No	4,100 (92.8)	1,824 (87.8)	
Missing	59 (1.1)	55 (2.1)	
Chronic lung disease			0.1
Yes	572 (10.7)	321 (13.1)	
No	3,889 (87.9)	1,801 (84.8)	
Missing	62 (1.3)	52 (2.1)	
Arthritis			<0.0001
Yes	1,222 (19.0)	812 (29.0)	
No	3,233 (79.5)	1,307 (68.7)	
Missing	68 (1.5)	55 (2.3)	
Depression or anxiety disorder			0.01
Yes	1,108 (24.7)	407 (19.3)	
No	3,336 (73.6)	1,705 (78.3)	
Missing	79 (1.7)	62 (2.4)	
Perceived benefits			
Track progress			<0.0001
Yes	1,956 (46.8)	223 (12.8)	
No	2,182 (46.9)	1,061 (50.9)	
Missing	62 (1.0)	84 (3.1)	
Not applicable	261 (4.4)	656 (27.6)	
Decision about treating illness			<0.0001
Yes	1,851 (42.1)	142 (7.7)	
No	2,277 (51.5)	1,131 (55.9)	
Missing	72 (1.1)	96 (3.2)	
Not applicable	262 (4.4)	665 (28.0)	
Self-efficacy			
Confidence in self-care			0.11
Completely confident	1,098 (23.4)	518 (26.5)	
Very confident	2,148 (46.4)	941 (41.0)	
Somewhat confident	1,068 (25.1)	557 (25.9)	
A little confident	127 (3.3)	89 (3.9)	
Not confident at all	42 (1.3)	37 (1.7)	
Missing	37 (0.6)	30 (1.0)	

Logistic regression results are shown in Table 3, Table 4. Due to the list-wise deletion of observations with missing data, the number of observations used in the regression models was n=4,930. As shown in model 1 (demographics only) of Table 3, the 18-34 age group was 2.2 times more likely to use electronic means to seek health information compared to the reference age group category (50-64 years; p=0.0007). Males were 27% less likely to use electronics to seek health information than females (p=0.02). Post-baccalaureate degree holders were three times more likely to use electronic means to seek health information compared to high school graduates (p<0.0001), and those earning $100,000 or more per year were 2.5 times more likely to use electronic means than the reference category ($0-19,999 per year; p=0.0003).

**Table 3:** Regression models predicting the use of electronic means for seeking health information, focusing on specific components of the Health Belief Model (n=4,930) Model 1 C-statistic: 0.762; Model 2 C-statistic: 0.541; Model 3 C-statistic: 0.594; Model 4 C-statistic: 0.742; Model 5 C-statistic: 0.511

Models	Odds ratio	P-value
Model 1		
Age (years)		
18-34	0.2	0.0007
35-49	1.6	0.004
50-64	Reference	
65-74	0.71	0.04
75+	0.26	<0.0001
Gender		
Male	0.73	0.02
Female	Reference	
Race/ethnicity		
Non-Hispanic white	Reference	
Non-Hispanic black	0.88	0.41
Hispanic	0.72	0.05
Non-Hispanic other	1.1	0.83
Education		
Less than high school	0.82	0.5
High school graduate	Reference	
Some college	1.87	<0.0001
Bachelor’s degree	3.1	<0.0001
Post-baccalaureate	3.13	<0.0001
Employment		
Employed	Reference	
Retired	0.86	0.39
Other	0.81	0.45
Household income		
$0-19,999	Reference	
$20,000-49,000	1.52	0.02
$50,000-99,999	1.98	0.008
$100,000 or more	2.48	0.0003
Model 2		
Perceived susceptibility		
General health		
Excellent	2.0	0.1
Very good	2.55	0.006
Good	2.11	0.03
Fair	1.54	0.19
Poor	Reference	
Model 3		
Perceived severity		
Diabetes/high blood sugar		
Yes	0.86	0.28
No	Reference	
Hypertension/high blood pressure		
Yes	0.75	0.007
No	Reference	
Heart condition		
Yes	0.71	0.11
No	Reference	
Chronic lung disease		
Yes	0.79	0.13
No	Reference	
Arthritis		
Yes	0.7	0.002
No	Reference	
Depression or anxiety disorder		
Yes	1.65	0.0003
No	Reference	
Model 4		
Perceived benefits		
Track progress		
Yes	3.5	<0.0001
No	Reference	
Decision about treating illness		
Yes	6.3	<0.0001
No	Reference	
Model 5		
Self-efficacy		
Confidence in self-care		
Completely confident	0.51	0.17
Very confident	0.64	0.36
Somewhat confident	0.55	0.24
A little confident	0.46	0.17
Not confident at all	Reference	

In terms of conceptual variables of the HBM, model 2, exploring perceived susceptibility (perceived health), showed that those who reported having very good health were 2.6 times more likely to use electronic means to seek health information (p=0.006). Model 3 focused on perceived severity and showed that those diagnosed with hypertension/high blood pressure were 25% less likely and those with arthritis were 30% less likely to seek health information than those who did not have these diagnoses; however, those diagnosed with depression or anxiety disorder were 65% more likely to use electronic means to seek health information compared to those who did not have the condition (p=0.0003). Model 4 focused on perceived benefits and concluded that respondents who felt that it was beneficial to use tablets to track progress on a health-related goal were 3.5 times more likely to use electronics to seek health information and those who intended to use it to make a decision about treating an illness were 6.3 times more likely to use electronic means to seek health information (p<0.0001). Model 5 showed that self-efficacy did not significantly predict the use of electronics to seek health information.

The receiver operator curve (ROC) was used to determine the true positive rate (sensitivity) and the false positive rate (specificity) of the HBM components in Table 3. C-statistics for perceived susceptibility (model 2) was 0.54, perceived severity (model 3) was 0.59, and perceived self-efficacy (model 5) was 0.5, which indicates that these models had no or weak predictive value for using electronic means to seek health information. However, the C-statistics value for perceived benefits (model 4) was 0.74, which indicates a good predictive model for using electronic means to seek health information.

Table 4 presents the full regression model, which showed that those who are 75 years of age or above are 63% less likely to use electronic means to seek health information compared to those who are 50-64 years of age. Those who had at least a bachelor’s degree were at least 3.4 times more likely to use electronic means to seek health information compared to high school graduates. Those with an income of $100,000 or more per year were 2.8 times more likely to use electronic means to seek health information (p<.0001). For the conceptual component of perceived severity, those who have depression were 42% more likely to use electronic means to seek health information, indicating that perceived severity predicted the use of electronics to seek health information at a higher rate. Those with higher perceived benefits because of their intent to track their health-related goals were 2.4 times more likely to use electronics to seek health information (p<0.0001), and those needing to make a decision about treating an illness were six times more likely to use electronic means to seek health information.

**Table 4:** Regression model for using electronic means to seek health information, selecting only significant findings from Table 3 (n=4,930)

Variables	Odds ratio	95% CI	P-value
Age (years)			
18-34	1.74	(1.06-2.85)	0.03
35-49	1.33	(0.93-1.92)	0.12
50-64	Reference		
65-74	0.88	(0.64-1.22)	0.44
75+	0.37	(0.27-0.51)	
Gender			
Male	0.93	(0.7-1.23)	0.62
Female	Reference		
Education			
Less than high school	0.82	(0.45-1.5)	0.52
High school graduate	Reference		
Some college	1.9	(1.45-2.47)	
Bachelor’s degree	3.39	(2.37-4.84)	
Post-baccalaureate	3.24	(2.24-4.69)	
Household income			
$0-19,999	Reference		
$20,000-49,999	1.63	(1.17-2.29)	0.004
$50,000-99,999	2.21	(1.47-3.33)	0.0001
$100,000 or more	2.82	(1.69-4.70)	
Perceived susceptibility			
General health			
Excellent	0.92	(0.31-2.68)	0.88
Very good	1.67	(0.65-4.28)	0.28
Good	1.98	(0.78-5.07)	0.15
Fair	2	(0.81-4.95)	0.14
Poor	Reference		
Perceived severity			
High blood pressure			
Yes	1.04	(0.81-1.34)	0.77
No	Reference		
Arthritis			
Yes	1.05	(0.79-1.38)	0.75
No	Reference		
Depression or anxiety			
Yes	1.42	(1.02-1.97)	0.04
No	Reference		
Perceived benefits			
Track progress			
Yes	2.38	(1.68-3.39)	
No	Reference		
Decision about treating illness			
Yes	6	(4.11-8.73)	
No	Reference		

## Discussion

Compared to previously published studies, HINTS respondents reported slightly lower electronic use specifically for health information (67.5% vs. 80%) [15], likely because the questions were framed specifically to using the internet to seek personal health information and not more generally, such as searching for self and others. Not surprisingly, non-Hispanic whites, younger respondents, those with higher income and education, and females were more likely to seek personal electronic health information. Similar to our findings, Robertson-Lang et al. (2011) have suggested that individuals with high education, high income, and those under the age of 65 are more likely to search for online health information, indicating the existence of a digital divide [15]. The digital divide separates people who have access to and use the internet from those who do not and parallels health disparity patterns in many other forms [16]. Renahy et al. (2010) indicated that online health information mostly benefits privileged individuals in terms of socioeconomic status, general health, and healthcare access. Inequalities in internet access create obstacles to taking full advantage of using the internet to seek health information. Individuals with low socioeconomic status, a minority race, older age, poor health, and those living in isolated geographical regions are less likely to have internet access [16].

With little additional descriptive information about electronic health information-seeking available, it was of interest that the most significant predictors were perceived severity and perceived benefits. However, not every serious diagnosis predicts more online use. Those who have depression were 42% more likely to use electronic means to seek health information, but those diagnosed with heart disease and arthritis were actually less likely to seek electronic health information. We also found that even controlling for demographics, individuals who use their tablets or smartphones to track progress on a health-related goal were 2.4 times more likely to use electronic means to seek health information. Moreover, users who find their tablets or smartphones helpful in making a decision about treating a health condition were six times more likely to use electronic means to seek health information. Using a tablet or phone suggest increased levels of comfort with electronic means, which likely affected respondents’ willingness to then also seek personal preventive and health information guidance. This aligns with other studies that found that one’s belief in the effectiveness of a proposed behavior predicts the likelihood of engaging in that behavior [17,18].

One of our most important findings is that those diagnosed with a mental health condition were 42% more likely to seek health information. This is likely due to the prevailing societal stigma about mental health, making the internet an attractive and seemingly safe way to seek information and guidance about their condition, which supports the finding [19] that people with depression or anxiety disorders prefer seeking online information in order to avoid talking about their problems or facing health providers and the finding [20] that people who are prone to anxiety may be more inclined to seek online health information. It is interesting, however, that individuals diagnosed with hypertension and arthritis were significantly less likely to seek health-related internet information, potentially a sign of their comfort in the treatment recommendations of their physicians, given the normalcy of these commonly diagnosed conditions and the age factor.

Ahadzadeh et al. (2015) collected data from educated, married women living in the state of Selangor, an urbanized state in Malaysia, who had internet access (n=293). The results showed that perceived health risk and health consciousness had a positive influence on health-related internet use. The perceived usefulness of online sources and attitudes toward internet use for health purposes partially facilitated the influence of health consciousness that could be gained from health-related internet use. The effect of perceived health risk on health-related internet use was fully mediated by the perceived usefulness of the internet and attitude towards its use. In the case of mental health diagnosis, our respondents may have perceived the internet to be more useful and necessary (given stigma and access challenges) than in the case of more mainstream diseases. Thus, engaging in health-related internet use is considered a proactive behavior rather than a reactive one [5]. Our findings mirrored this study as individuals who showed the perceived health risk of depression/anxiety and who found benefits in using electronic means to seek health information by tracking progress on a health-related goal or making a decision in treating illness were more likely to search online for health information.

Kim and Park (2012) conducted a study to develop a model to describe the behavioral intention and the health behavior of health-related information technology consumers. They developed a cross-sectional descriptive correlational design, using the Technology Acceptance Model (TAM) with additional mediating variables from the HBM and the Theory of Planned Behavior. The results showed that perceived threat, perceived usefulness, and perceived ease of use significantly affected users’ health-related attitude and behavioral intention. An Individual’s health condition, health belief, and health information technology self-efficacy had a significant impact on attitude and behavioral intention through the mediators of perceived threat, usefulness, and ease of use [18]. Their study confirms our findings as respondents who found perceived benefits of using electronics were significantly more likely to use electronic means to seek health information for a disease that may have represented a more personal threat so that they sought treatment options or that they were reluctant to share given stigma.

The level of understanding of patient health information may be a predictor of health behavior and the use of health services. Individuals who frequently seek health information tend to participate in their care management. Informed patients use the internet as a useful informational tool to gain an understanding of symptoms and medical conditions or read online health interventions [21]. For instance, Tate et al. (2015) encourage using online sources to participate in physical activity interventions that aim at reducing body weight by increasing motivation levels [22]. Internet-based exercise interventions that provide exercise coaching and goal-setting can result in increased physical activity engagement [23]. Our findings provide a better understanding of people’s behavior, which can help in improving online health interventions.

Due to the cross-sectional nature of the study, it was not possible to infer causal relationships between variables in the study. The response rate for both datasets was 32%, which is considered relatively low and may have created a non-response bias. In addition, using existing datasets and merging two datasets limited the choices related to variables. Since HINTS was not optimized for HBM research, we could only do our best to align available variables to the model. This may be the reason that, contrary to the model prediction, we found only the perceived severity and perceived benefits components of the HBM to be significantly related to our outcome. However, given the increasing importance of internet-based health information-seeking, more research exploring theory-guided profiles of those availing themselves of this approach is needed.

## Conclusions

Our findings suggest targeted framing of online health information, as those with plans for preventive behavior, those facing a serious illness, and those with mental health needs are more likely to use the internet for health-specific information. As the internet remains largely unregulated, with few quality controls, it is critical that health systems link their patients to trustworthy resources for preventive information as well as treatment-related information, since many already engage in internet-guided health information-seeking.

Seeking health information online is a relatively new approach for gaining health knowledge, changing health behaviors, and improving health outcomes. As research continues, there is a need to conduct further studies to better understand and evaluate individuals’ use of the internet and social media to look for health information. The results of the study can create an opportunity for policymakers to develop policies for more effective online health interventions that can help in changing health behaviors. Linking health systems to online platforms can ensure disseminating reliable online health information by trustworthy sources, which could contribute to improving health behaviors among adults in the United States in a cost-effective manner.
